# Parental Mental Health and Child Maltreatment in the COVID-19 Pandemic: Importance of Sampling in a Quantitative Statistical Study

**DOI:** 10.2196/52043

**Published:** 2025-01-24

**Authors:** Lara Engelke, Claudia Calvano, Steffi Pohl, Sibylle Maria Winter, Babette Renneberg

**Affiliations:** 1 Clinical Psychology and Psychotherapy Department of Education and Psychology Freie Universität Berlin Berlin Germany; 2 Clinical Child and Adolescent Psychology and Psychotherapy Department of Education and Psychology Freie Universität Berlin Berlin Germany; 3 German Center for Mental Health (DZPG) Berlin Germany; 4 Division Methods and Evaluation Department of Education and Psychology Freie Universität Berlin Berlin Germany; 5 Department of Child and Adolescent Psychiatry, Psychosomatics and Psychotherapy Charité - Universitätsmedizin Berlin, Corporate Member of Freie Universität Berlin and Humboldt-Universität zu Berlin Berlin Germany

**Keywords:** COVID-19, parental stress, parental mental health, child maltreatment, data collection methods, web-based surveys, convenience sample, sampling methods

## Abstract

**Background:**

Results on parental burden during the COVID-19 pandemic are predominantly available from nonrepresentative samples. Although sample selection can significantly influence results, the effects of sampling strategies have been largely underexplored.

**Objective:**

This study aimed to investigate how sampling strategy may impact study results. Specifically, we aimed to (1) investigate if outcomes on parental health and child maltreatment during the COVID-19 pandemic from a convenience sample differ from those of a specific representative sample and (2) investigate reasons for differences in the results.

**Methods:**

In 2020, we simultaneously conducted 2 studies: (1) a web-based survey using a convenience sample of 4967 parents of underage children, primarily recruited via social media, and (2) a study using a quota sample representative of the German adult population with underage children (N=1024), recruited through a combination of telephone interviews and computer-assisted web interviews. In both studies, the same questionnaire was used. To evaluate the impact of sampling, we compared the results on outcomes (parental stress, subjective health, parental mental health, general stress, pandemic-related stress, and the occurrence of child maltreatment) between the 2 samples. To explain differences in the results between the 2 studies, we controlled for sociodemographic data, parent-related risk factors, and COVID-19–related experiences.

**Results:**

Compared to parents from the quota sample, parents from the convenience sample reported significantly more parental stress (η^2^=0.024); decreased subjective health (η^2^=0.016); more anxiety and depression symptoms (η^2^=0.055); more general stress (η^2^=0.044); more occurrences of verbal emotional abuse (VEA; φ=0.12), witnessing domestic violence (WDV; φ=0.13), nonverbal emotional abuse (NEA; φ=0.03), physical abuse (φ=0.10), and emotional neglect (φ=0.06); and an increase of child maltreatment (VEA: exp(B)=2.95; WDV: exp(B)=3.19; NEA: exp(B)=1.65). Sociodemographic data, parent-related risk factors, and COVID-19–related experiences explained the differences in parental stress (remaining difference between samples after controlling for covariates: η^2^=0.002) and subjective health (remaining difference between samples after controlling for covariates: η^2^=0.004) and partially explained differences in parental mental health (remaining: η^2^=0.016), general stress (remaining: η^2^=0.014), and child maltreatment (remaining: VEA: exp(B)=2.05 and WDV: exp(B)=2.02) between the 2 samples. The covariates could not explain the difference in NEA (exp(B)=1.70). We discuss further factors that may explain the unexplained differences.

**Conclusions:**

Results of studies can be heavily impacted by the sampling strategy. Scientists are advised to collect relevant explaining variables (covariates) that are possibly related to sample selection and the outcome under investigation. This approach enables us to identify the individuals to whom the results apply and to combine findings from different studies. Furthermore, if data on the distribution of these explanatory variables in the population are available, it becomes possible to adjust for sample selection bias.

## Introduction

### Background

The COVID-19 pandemic and the resulting consequences (eg, restrictions, such as school and daycare closings, social distancing, and stay-at-home orders) posed various challenges, especially for families. Parents felt stressed [1-4] and were especially burdened during the COVID-19 pandemic [1,5,6]. Studies showed worsening or significant impairments of parental mental health (depression, anxiety, and stress) [2,6-16], a significant increase in parental stress [3,14,17-20], and an increase in the occurrence of child maltreatment during the COVID-19 pandemic [17,21,22].

The existing trend toward convenience sampling in psychological research [23-25] increased during the COVID-19 pandemic [26]. As is the case for most studies on mental burden and well-being of families during the COVID-19 pandemic, findings on parental outcomes are primarily generated through web-based studies with convenience sampling (eg, [4,7,10,13-17,19,22,27,28]). In a web-based study, convenience sampling is often conducted by recruiting participants through easily accessible methods, such as social media posts, previous study participants, or email lists. Consequently, they are often not representative of the population of interest. Only a few studies evaluating the impact of the COVID-19 pandemic on families recruited a representative sample (eg, [1-3,11]). Therefore, many studies are not representative of the general population or the specific population of interest. In other words, in web-based convenience samples, the general population is often not adequately represented. Differences in sample characteristics may not be a problem, if the sample characteristics that differ are irrelevant for the outcome one wants to assess. However, if the samples differ in variables that are relevant for the outcomes (eg, single parents are less likely to take part in a web-based convenience study on parental burden because they may not have the time; at the same time, single parents will more likely be affected by COVID-19 restrictions), the results will be biased and cannot be generalized to the overall population.

Studies have shown substantial differences in sociodemographic composition from web-based convenience samples in comparison with representative samples [29-32]. In their systematic literature review on recruitment via Facebook for health-related studies, Whitaker et al [33] found that some studies showed an overrepresentation of White, female, younger individuals with higher education and higher income. The overrepresentation of these sample characteristics in web-based convenience samples on parenting and family research is also known [34-39]. In various web-based convenience samples assessing the impact of the COVID-19 pandemic on parents, participants are more likely to be female [4,9,14-17,19,22,27,40-45], in a relationship [4,15,19,22,40,41,43], and have higher education [4,14,16,17,27,41-43,45-47]. Differences in sociodemographic variables (eg, sex, child age, parental age, and socioeconomic status) compared to the general population further influence the outcomes, as some of these factors are associated with poorer parental outcomes and greater family burden during the pandemic [8,11,16,40-42,48-60]. Thus, these study results may not necessarily generalize to the overall population during the COVID-19 pandemic [26,61-66].

The generalizability of results from nonprobability studies with web-based convenience samples was already discussed before the COVID-19 pandemic [29,31,67-69]. In their literature review, Cornesse et al [31] concluded that the generalizability of probability sample surveys (eg, representative samples) is higher than that of nonprobability sample surveys (eg, web-based convenience samples). Similar results were found by other researchers [25,30,32,70-76]. Even before the COVID-19 pandemic, studies assessing mental health indicated that individuals with higher burden were more likely to choose to participate in web-based convenience sample studies, resulting in discrepancies in burden scores compared to representative samples [25,77,78]. This selection effect is also evident in studies assessing the impact of the COVID-19 pandemic, resulting in an overestimation and underestimation of specific outcomes [65,79].

Studies on the generalizability of web-based convenience sampling in parenting and family research are scarce. One Australian study found that mothers surveyed on the web via Facebook, compared to a population-representative sample, showed sociodemographic differences (eg, overrepresentation of younger individuals, those in a relationship, with higher education, and who spoke only English at home) as well as poorer mental health and self-rated health [39]. Similarly, Bennetts et al [80] reported higher psychological distress in their parent sample recruited through Facebook compared to a representative sample.

### Objective

To the best of our knowledge, no published studies have investigated the generalizability of results from web-based convenience samples on parental outcomes during the COVID-19 pandemic, leaving the potential biasing effect unknown. The following research questions were addressed: (1) Are results from a convenience sample during the COVID-19 pandemic comparable to results from a specific representative sample regarding parental outcomes (ie, parental stress, parental health, and the occurrence of child maltreatment)? and (2) To what extent can we explain differences in outcomes between the studies by sample characteristics?

## Methods

### Study Design

This study is based on the project “Parenting in Pandemic Times.” The target population of the project was parents with underage children in the household in Germany (national) with sufficient German language skills. Data presented in this paper refer to 2 separate studies conducted simultaneously. First, a quota sample was collected by the market research company INFO Marktforschungsinstitut between August 3 and August 11, 2020. Computer-assisted telephone interviews (CATIs; n=402) were combined with a computer-assisted web interview (CAWI; n=622) to provide data that are as representative as possible. The CAWI allowed respondents to complete the questionnaire on the web at home on a computer, tablet, cell phone, or other smart device. A dual-frame design was used for the CATI sample to reach respondents via landline and cell phone. Participants in the CAWI sample were recruited from an active web-based access panel of the market research institute. They received incentives for their participation. In the first step, specific representativity was assured by recruiting participants based on quota obtained from the latest microcensus data regarding parental sex, parental age, number of children, federal state, school educational level (with vs without A-level), and income. In the second step, to adjust for the remaining disproportion in these variables due to unit nonresponse, INFO Marktforschungsinstitut additionally used poststratification weighting. While educational level was assessed in 4 categories, representativeness (eg, quota and weighting) was only ensured for the dichotomous variable of having an A-level (high education) versus not having an A-level. For the dichotomous variable on education, specific representativity could be assured. The weighting took place retrospectively to the actual quota sampling. (For more information, refer to the study by Calvano et al [2]; in this study, weighting only slightly altered the strata-variable distribution in the specific representative study. Calvano et al [2] determined that using unweighted as compared to weighted data does not alter the findings or conclusions.) Second, a convenience sample was collected between August 2 and September 16, 2020. The recruitment of this sample (N=4967) took place through requests and emails via an web link through social media (eg, Facebook groups, Instagram, and Twitter; 2561/4967, 51.56%; refer to Multimedia Appendix 1, picture 1), friends and family (769/4967, 15.48%), internet (658/4967, 13.25%), schools (551/4967, 11.09%), daycare facilities (263/4967, 5.29%), work (84/4967, 1.69%), clubs (65/4967, 1.31%), clinics for child and adolescent psychiatry (34/4967, 0.68%), child and adolescent therapists and pediatricians (18/4967, 0.36%), newspapers (32/4967, 0.64%), and child protection outpatient clinics (16/4967, 0.32%). As the research team was based in Berlin, Germany, recruitment primarily took place in Berlin through schools, daycare facilities, child and adolescent therapists and physicians, clubs, and newspapers. Social media requests were placed in groups with German-speaking parents. A total of 42.5% (2111/4967) of the convenience sample came from Berlin. Participants in the convenience sample were able to take part in a raffle for vouchers and iPads at the end of the survey. In both samples, few data were excluded due to an unrealistic short completion time (convenience: 53/5020, 1.06%; specific representative: 66/1090, 6.06%). Noteworthily, the surveys were identical in content, that is, all measures and instructions applied were identical.

### Ethical Considerations

Data collection, analysis, evaluation, and reporting were conducted in accordance with the current ethical guidelines and data protection laws. All data are anonymized. The Ethics Committee of Charité – Universitätsmedizin Berlin (EA2/128/20; July 27, 2020) approved the study. Participants received information on the purpose of the research, the scope and duration of the diagnostic measures, possible risks and benefits, the voluntary nature of participation, and all aspects of data protection. For participation in the study, participants agreed to an informed consent declaration. Participants were provided with contact details for support services. Participants in the convenience sample could take part in a prize raffle for cash vouchers. Participants in CAWI received incentives from the market research company.

### Measures

We distinguished between explaining variables (sociodemographic data, parent-related risk factors, and pandemic-related experience) and parental outcomes (parental stress, subjective health, parental mental health, general stress, and child maltreatment).

#### Explaining Variables

##### Sociodemographic Data

Data on parental sex and age, parental status (biological parent, stepparent, and other), nationality, number of children in the household, children’s age and sex, marital status, federal state, parental school and professional education, and parental current employment status were assessed. We calculated the Winkler Index [81] with reference data from the German population to classify the socioeconomic status as low, medium, or high.

##### Parent-Related Risk Factors

The alcohol abuse module of the Patient Health Questionnaire, German version [82,83] was applied to assess parents’ risk of alcohol abuse during the COVID-19 pandemic. Furthermore, the study assessed parents’ experiences of sexual or physical violence in adulthood, their own history of childhood sexual or physical abuse, and the presence of mental disorders. In addition, parents were asked to report the presence of a severe or chronic physical condition and if they belonged to the risk group for COVID-19.

##### Pandemic-Related Experience

To measure pandemic-related experiences and stress related to COVID-19, the Pandemic Stress Scale (Winter, S, Corona Belastungsskala. Charité - Universitätsklinikum Berlin, Klinik für Psychiatrie, Psychosomatik und Psychotherapie des Kindes- und Jugendalter, Sektion Traumafolgen und Kinderschutz, unpublished data, January 2020) was developed. Questions were answered with respect to household members, the family, or the parents themselves. At first, COVID-19–related personal experiences were assessed, that is, contact with persons with a COVID-19 infection, hospital admission, or death. Afterward, the month or months with the subjectively highest burden (January 2020 to August 2020) were assessed.

#### Parental Outcomes

##### Parental Stress

To assess negative and positive perceptions of parenthood, the Parental Stress Scale [84] was implemented for (1) the time with the subjectively highest burden (total sample [convenience and specific representative sample combined]: Cronbach α=0.88) and (2) before the COVID-19 pandemic, that is, January 2020 in retrospect (total sample: Cronbach α=0.86). Parents rated their perception on the 18 items on a 5-point frequency scale. Higher scores expressed higher parental stress.

##### Subjective Health

To measure parents’ perception of their own health (1) at the time of the highest burden and (2) retrospectively in January 2020, a well-established single-item measure [85] with a 10-point scale was implemented. Higher scores indicated better self-rated health.

##### Parent Mental Health

To assess depression and anxiety symptoms, the Patient Health Questionnaire 4 [86] was applied (total sample: generalized anxiety, Cronbach α=0.70; depression, Cronbach α=0.68; and total score, Cronbach α=0.78). Higher scores on the 4-point scale indicated poorer mental health.

##### Pandemic-Related Stress

The Pandemic Stress Scale (Winter, S, Corona Belastungsskala. Charité - Universitätsklinikum Berlin, Klinik für Psychiatrie, Psychosomatik und Psychotherapie des Kindes- und Jugendalter, Sektion Traumafolgen und Kinderschutz, unpublished, January 2020) consists of 14 items about specific restrictions (eg, daycare closings and social distancing), which parents rated regarding their subjective burden on a 5-point scale (ranging from not at all burdening to very burdening). The answering format also included a “not applicable” option. The Pandemic Stress Scale refers to the time point with the subjectively highest burden during the COVID-19 pandemic so far, from the beginning to August 2020. The scale had good internal consistency (total sample: Cronbach α=0.85). Higher scores expressed a higher subjective burden.

##### General Stress

To measure general parental stress at the time of the subjectively highest burden, we implemented the stress module of the Patient Health Questionnaire, German version [83] (total sample: Cronbach α=0.71). It consists of 10 items with several psychosocial stressors on a 3-point scale, and higher scores indicated higher general stress.

##### Child Maltreatment

Parents provided information about child maltreatment regarding the children in their household. The Pediatric Maltreatment and Abuse Chronology of Exposure interview [87-89] was adjusted to collect data on household dysfunction, child neglect, and abuse. First, parents were asked if the children ever faced severe stressful life experiences (eg, abuse, violence, and neglect). Afterward, 10 items on distinct subtypes of child maltreatment experiences were asked: 5 items on different types of child abuse (nonverbal emotional abuse [NEA], verbal emotional abuse [VEA], physical abuse, witnessing domestic violence [WDV], and sexual abuse), 3 items on different types of neglect (supervisory neglect, physical neglect, and emotional neglect), and 2 items on different types of household dysfunction (problems regarding mental illness or alcohol or substance use in the household). Only the first item explicitly referred to severe levels of maltreatment; the other items on subtypes mirrored comparatively lower severity levels in our surveys (according to the maltreatment classification system [90,91]). First, a dichotomous yes or no item was presented for each subtype asking parents if the children ever experienced a specific subtype of child maltreatment. If the answer was yes, a follow-up question was posed, asking about the change in frequency since the beginning of the COVID-19 pandemic, with response options on a 5-point scale ranging from “significantly more often” to “significantly less often.” For more details about the measure, refer to the study by Calvano et al [2]. To simplify the presentation of the results, we dichotomized the responses on child maltreatment during the COVID-19 pandemic into increase (increase or significant increase) versus no increase (staying the same, decrease, or significant decrease). To provide meaningful results, we decided not to further compare subtypes with case numbers smaller <30.

### Data Analysis

For investigating the research questions, we aimed to answer the following: To what extent do the 2 studies differ with respect to the parental outcomes and specific sample characteristics? How well can we predict sample membership by these sample characteristics? How well can we explain differences in parental outcomes between the 2 samples by sample characteristics?

#### Investigating Differences in Explaining Variables Between Samples

We calculated effect sizes describing the differences in explaining variables/covariates (sociodemographic data, parent-related risk factors, and COVID-19–related experiences) between the 2 studies. The significance of differences between the samples was investigated using 2-tailed *t* tests or Welch tests for interval-scaled variables (eg, age, rooms in the household, parental mental health, and parental stress) and chi-square tests for nominal and ordinal variables (eg, sex, federal state, nationality, garden, belonging to a risk group for COVID-19, and school education). For metric variables, we report Cohen *d,* and for nominal and ordinal-scaled variables, we report the phi coefficient.

After examining differences between studies for each individual variable, the next step was to consider all covariates simultaneously. As such, we used logistic regression to predict sample membership (0=specific representative sample and 1=convenience sample) by the covariates, that is, sociodemographic data, parent-related risk factors, and COVID-19–related experience. Due to estimation requirements, we only reported on the prediction of study membership for categories of categorical outcomes with an absolute frequency >30.

#### Investigating Differences in Parental Outcomes Between Samples

We calculated effect sizes describing the differences in outcomes between the 2 studies, using ANOVA. We report partial η^2^ for effect size. Chi-square tests were used for nominal and ordinal outcomes, with the phi coefficient reported for effect size.

#### Explaining Differences in Parental Outcomes Between Samples

We conducted analyses of covariance (ANCOVAs) using sample type and all covariates as predictors for the respective outcome variable. We then compared the adjusted effect estimates for sample type from the ANCOVA with the unadjusted effect estimates from an ANOVA that did not include any covariates. When in the ANCOVA the equality of error variances assumption was violated, we calculated parameter estimates with robust standard errors for the ANCOVAs. We could not find any considerable differences in the results, and therefore, we decided to present the results of the common and well-known standard methods. The size of the adjusted difference in outcomes between types of samples (adjusted effect estimate) indicates how much the covariates describe the difference in study results between the 2 samples. If effect estimates of the type of sample on the outcome variable decrease or even disappear after controlling for covariates, the considered covariates explain partly (in case of just decrease) or fully (in case of disappearance) the differences between samples for the respective outcome. Herein, we compared the effect sizes (η^2^) of the sample indicator using ANOVA, with the covariate-adjusted effect sizes (η^2^) of the sample indicator from the ANCOVA.

For an increase in child maltreatment (0=no increase and 1=increase), binary logistic regressions for predicting each subtype of child maltreatment from study type (and covariates) were used. Odds (exp(B)) of the type of sample adjusted for all covariates were compared to unadjusted odds (exp(B)) with study type being the only predictor. If the odds of sample type influencing the outcome variable decrease or approach 1 after controlling for covariates, it indicates that the covariates partially (if the odds decrease) or fully (if the odds approach 1) explain the differences between the samples for the respective outcome. Due to estimation requirements, we only reported on covariate-adjusted outcome differences between studies for categories of categorical outcomes with an absolute frequency >30. All analyses were performed using SPSS (version 29; IBM Corp).

## Results

In the following, the results of the convenience sample are compared with the results of the specific representative sample. Note that results are hardly impacted by the weighting [2].

### Investigating Differences in Sample Characteristics

We evaluated differences in sociodemographic data, parent-related risk factors, and COVID-19–related experiences.

#### Sociodemographic Data

Tables 1 and 2 depict the comparison of sociodemographic characteristics of the 2 samples (convenience vs specific representative). In the convenience sample, 4967 people, primarily women (n=4349, 87.56%), participated with a mean age of 39.11 (SD 6.25; range 20-70) years. The average number of children in the household was 1.82 (SD 0.78; range 1-11). These children were on average aged 6.46 (SD 4.25; range 0-17) years. In 42.5% (2111/4967) of the convenience sample, the families lived in Berlin. The distribution of the participants among the federal states and a comparison between the samples is shown in Table S1 in Multimedia Appendix 1. In most cases, participants worked either part time (2051/4967, 41.29%) or full time (1778/4967, 35.8%). Most participants were highly educated with a high school education (4305/4967, 86.67%) or a university degree (3390/4967, 68.25%), and they had a high socioeconomic status (3165/4967, 63.72%).

The specific representative sample comprised 1024 participants (n=534, 52.14% women) with a mean age of 40.89 (SD 8.17; range 18-73) years. On average, 1.69 (SD 0.77; range 1-6) children aged <18 years lived in the household. The mean age of the children was 8.92 (SD 4.92; range 0-17) years. In total, 4.3% (44/1024) of the participants lived in Berlin. In 55.66% (570/1024) of the cases, the parent worked full time, and in 27.64% (283/1024) of the cases, they worked part time. Most participants had a middle school education (480/1024, 46.88%), had completed an apprenticeship (586/1024, 57.23%), and had a middle socioeconomic status (585/1024, 57.13%).

**Table 1:** Sociodemographic characteristics of the convenience sample and the specific representative sample—parent data.

		Convenience sample 2020, (N=4967), n (%)	Specific representative sample 2020^a^, (N=1024), n (%)	Comparison, test statistics		*P* value		Effect size	
Parental sex (female), n (%)		4349 (87.6)	534 (52.1)	*χ*^*2*^_1_=706.2		<.001		φ=0.34	
Biological parent, n (%)		4913 (97.8)	979 (95.6)	χ^2^_1_=57.1		<.001		φ=0.10	
Single parent, n (%)		504 (10.1)	123 (12)	*χ*^*2*^_1_=3.2		.08		φ=0.02	
**Nationality, n (%)**									
	German	4803 (94.7)	1001 (97.8)	*χ*^*2*^_1_=3.1		.08		φ=0.02	
	Other	270 (5.3)	23 (2.2)	*χ*^*2*^_1_=16.3		<.001		φ=0.05	
Age (years), mean (SD)		39.11 (6.25)	40.89 (8.17)	t_1218.10_=6.58		<.001		*d*=0.27	
Marital status: in a relationship, n (%)		4568 (92.9)	917 (89.6)	*χ*^*2*^_1_=13.3		<.001		φ=0.05	
Federal State of Germany: city-state, n (%)		2263 (45.6)	75 (7.3)	*χ*^*2*^_1_=521.6		<.001		φ=0.30	
**School education, n (%)**									
	Low (up to 9 years of schooling)	58 (1.2)	109 (10.7)	*χ*^*2*^_1_=281.4		<.001		φ=–0.22	
	Middle (10 years of schooling)	564 (11.3)	480 (42.5)	*χ*^*2*^_1_=744.4		<.001		φ=–0.35	
	High (up to 13 years of schooling)	4305 (86.7)	426 (41.6)	*χ*^*2*^_1_=1038.4		<.001		φ=0.42	
	No or other education	40 (0.7)	9 (5.2)	*χ*^*2*^_1_=0.1		.81		φ=–0.003	
**Professional education, n (%)**									
	Apprenticeship	1041 (21.0)	586 (57.2)	χ^2^_1_=564.5		<.001		φ=–0.31	
	Technical school	308 (6.2)	119 (11.6)	χ^2^_1_=37.7		<.001		φ=–0.08	
	University	3390 (68.3)	247 (24.1)	χ^2^_1_=693.1		<.001		φ=0.34	
	No or other education	228 (4.6)	72 (7)	χ^2^_1_=10.6		<.001		φ=–0.04	
Current employment status: working, n (%)		3983 (80.2)	863 (84.3)	χ^2^_1_=9.2		<.001		φ=–0.04	
**Socioeconomic status index** ^b^ **, n (%)**					t_1268.97_=–4.05		<.001		*d*=–.17
	Low	116 (2.3)	100 (9.8)						
	Middle	1686 (33.9)	585 (57.5)						
	High	3165 (63.7)	338 (33)						
**Household**									
	Balcony, n (%)	3125 (62.9)	598 (58.4)	χ^2^_1_=7.4		.007		φ=–0.04	
	Garden, n (%)	2969 (59.8)	768 (75.0)	χ^2^_1_=83.9		<.001		φ=–0.12	
	Rooms, mean (SD)	*4.31 (3.13)*	*4.87 (4.42)*	t_1242.91_=3.84		<.001		*d*=0.16	
	Other persons in the household, n (%)	4357 (87.7)	914 (89.3)	χ^2^_1_=1.9		.17		φ=–.02	

^a^Weighted data.

^b^Index calculated according to the Winkler Index [81].

**Table 2:** Sociodemographic characteristics of the convenience sample and the specific representative sample—child data.

		Convenience sample 2020 (N=4967), n (%)	Specific representative sample 2020^a^ (N=1024), n (%)	Comparison, test statistics		*P* value		Effect size	
**Number of children**					t_1492.82_=–4.90		<.001		*d*=–0.17
	1	1813 (36.65)	474 (46.3)						
	2	2417 (48.7)	427 (41.7)						
	≥3	737 (14.8)	122 (12)						
**Child’s age (y)**					t_1329.24_=18.06		<.001		*d*=0.70
	0-2	1953 (39.3)	230 (22.3)						
	3-5	2380 (48)	283 (27.4)						
	6-12	3634 (73.1)	713 (69.6)						
	13-17	1046 (21.1)	506 (49.5)						
Child’s sex (female)		4387 (48.7)	862 (49.9)	—^b^		—		—	
Children living in 2 households		155 (3.1)	27 (2.6)	*χ*^*2*^_1_=0.7		.41		φ=–.01	
Previous contact with social and family services		1197 (24.1)	249 (24.3)	*χ*^*2*^_1_=0.2		.88		φ=–.01	

^a^Weighted data.

^b^Not applicable.

The 2 samples differed substantially in relevant characteristics. The convenience sample had a significantly higher number of women, the children were younger, participants were more frequently from a city-state (ie, Berlin), and had a higher school and professional education than the specific representative sample (all differences with medium effect sizes). Compared to the specific representative sample, in the convenience sample, we observed small differences in the following variables: participants were significantly younger, had more children aged <18 years in the household, had a higher socioeconomic status, were of another nationality than German, were more frequently biological parents and in a relationship, had fewer rooms in the household, and had less frequently a garden. Samples were comparable regarding being a single parent, child sex, current employment status, having a balcony, children living in 2 households (eg, in case of separated parents), and having others in the household or previous contact with social family services.

#### Parent-Related Risk Factors

Table 3 shows parent-related risk factors. Significantly fewer parents from the convenience sample reported belonging to the risk group for severe COVID-19. A significantly higher share of parents from the convenience sample reported a current or previous mental disorder and a history of own child abuse or neglect. Furthermore, a significantly higher share of parents from the convenience sample reported March and April 2020 as the most stressful months. In contrast, a significantly higher share of parents from the specific representative sample reported that “all months were equally stressful” and “no month was especially stressful.” The effect-sizes were small.

**Table 3:** Parent-related risk factors: descriptive data and comparison between the samples.

	Convenience sample 2020 (N=4967), n (%)	Specific representative sample 2020^a^ (N=1024), n (%)	Comparison		
			*χ*^*2*^ (*df*)	*P* value	φ
Parental risk of alcohol abuse^b^	317 (6.38)	56 (5.47)	1.2 (1)	.27	0.01
Parental mental disorder	864 (17.39)	107 (10.45)	30.2 (1)	<.001	0.07
Chronic severe health condition	604 (12.16)	146 (14.26)	3.4 (1)	.07	–.02
Parental history of child abuse or neglect	1562 (31.45)	238 (23.24)	27.0 (1)	<.001	0.07
Parental experience of violence in adulthood	601 (12.10)	114 (11.13)	0.8 (1)	.39	–.01

^a^Weighted data.

^b^n=3430 (convenience sample) and n=700 (specific representative sample) parents indicated that they regularly drink alcohol. n=317 (convenience sample) and n=56 (specific representative sample) were at risk for alcohol abuse according to Patient Health Questionnaire-D [83].

#### COVID-19–Related Experiences

Table 4 shows the COVID-19–related experiences. In both samples, nearly a quarter of the participants reported reduced working hours (convenience sample: 1197/4967, 24.1% and specific representative sample: 277/1024, 27.05%), and one-fifth of the participants reported a significant financial loss due to COVID-19 (convenience sample: 1111/4967, 22.37% and specific representative sample: 221/1024, 21.58%). In addition, April 2020 (convenience sample: 3508/4967, 70.62% and specific representative sample: 487/1024, 47.56%) and May 2020 (convenience sample: 2975/4967, 59.89% and specific representative sample:430/1024, 42%) were the most stressful months until August 2020 in both samples.

**Table 4:** COVID-19–related experiences: descriptive data and comparison.

		Convenience sample 2020 (N=4967), n (%)	Specific representative sample 2020^a^ (N=1024), n (%)	Comparison		
				*χ^2^(df)*	*P* value	φ
**Effects of the COVID-19 pandemic on the health situation**						
	Family or household member infected with COVID-19	93 (1.87)	22 (2.15)	0.3 (1)	.56	–0.01
	A family or household member was hospitalized due to COVID-19	15 (0.30)	8 (0.78)	5.0 (1)	.02	–0.03
	Family or household member passed away due to COVID-19	13 (0.26)	4 (0.39)	0.5 (1)	.48	0.01
Parent belongs to the risk group for severe COVID-19		328 (6.60)	103 (10.06)	15.3 (1)	<.001	0.05
**Effects of the COVID-19 pandemic on job situation**						
	Reduced working hours	1197 (24.10)	277 (27.05)	4.0 (1)	.046	0.03
	Job loss	235 (4.73)	55 (5.37)	0.8 (1)	.39	0.01
	Significant financial loss	1111 (22.37)	221 (21.58)	0.3 (1)	.58	–0.01
**Most stressful months**						
	February	113 (2.28)	21 (2.05)	0.2 (1)	.66	0.01
	March	2207 (44.43)	268 (26.17)	116.8 (1)	<.001	0.14
	April	3508 (70.63)	487 (47.56)	203.3 (1)	<.001	0.18
	May	2975 (59.90)	430 (41.99)	110.9 (1)	<.001	0.14
	June	1634 (32.90)	263 (25.68)	20.4 (1)	<.001	0.06
	July	582 (11.72)	117 (11.43)	0.1 (1)	.79	0.003
	August	307 (6.18)	46 (4.49)	4.4 (1)	.04	0.03
	All months were equally stressful	288 (5.80)	125 (12.21)	54.3 (1)	<.001	–0.10
	No month was especially stressful	381 (7.67)	186 (18.16)	109.1 (1)	<.001	–0.14

^a^Weighted data.

All 34 covariates were simultaneously considered in a binomial logistic regression for the prediction of sample membership (0=specific representative sample and 1=convenience sample). Explained variance in outcomes by the predictors was large (Nagelkerke *R*^2^=0.56) and statistically significant from 0 (χ^2^_36_=2411.4; *P*<.001). The overall accuracy percentage in classification was 89.6%, with a sensitivity of 96.3% and a specificity of 57.2%. The 5 most important predictors for convenience sample membership were as follows: living in a federal state in Germany (Berlin, Bremen, or Hamburg; odds ratio [OR] 14.75, 95% CI 10.93-19.91), parental sex female (OR 11.13, 95% CI 8.89-13.94), parental mental disorder (OR 2.18, 95% CI 1.63-2.92), being a biological parent (OR 2.05, 95% CI 1.11-3.79), and higher education (professional education [university]: OR 2.02, 95% CI 1.33-3.08 and school education [high school]: OR 2.01, 95% CI 0.77-5.23). All model coefficients and odds ratios can be found in Table S2 in Multimedia Appendix 1.

### Investigating Differences in Parental Outcomes Between Samples

Differences in parental outcomes between the convenience sample and the specific representative sample are displayed in Table 5 for parental outcomes and Tables 6 and 7 for child maltreatment outcomes.

**Table 5:** Descriptive data: investigating and explaining differences between the convenience and the specific representative samples in parental outcomes.

	Convenience sample (N=4967), mean (SD)^a^	Specific representative sample^b^ (N=1024 [weighted]), mean (SD)^a^	Comparison between the samples, ANOVAs (unadjusted)				ANCOVAs^c^ (adjusted)		
			*F* test (*df*)	*P* value	η^2^	*F* test (*df*)		*P* value	η^2^
Parental stress: January 2020	36.07 (8.67)	34.72 (10.63)	18.35 (1)	<.001	0.003	0.33^d^		.56	<0.001
Parental stress: time of the highest burden	41.37 (10.50)	36.93 (10.45)	149.97 (1)	<.001	0.024	14.92^e^		<.001	0.002
Subjective health: January 2020	7.36 (1.76)	7.34 (2.04)	0.03 (1)	.87	<0.001	0.06^f^		.81	<0.001
Subjective health: time of the highest burden	6.06 (2.27)	6.80 (2.21)	101.13 (1)	<.001	0.016	21.69^g^		<.001	0.004
Patient Health Questionnaire-4 (anxiety and depression symptoms)	4.37 (3.0)	2.52 (2.63)	354.94 (1)	<.001	0.055	97.98^h^		<.001	0.016
General stress	7.40 (3.73)	5.28 (4.13)	4023.2 (1)	<.001	0.044	84.21^i^		<.001	0.014
Pandemic-related stress	31.80 (8.03)	31.97 (10.96)	0.56 (1)	.45	<0.001	6.75^j^		.009	0.001

^a^Unadjusted significant covariates.

^b^Weighted data.

^c^ANCOVA: analysis of covariance.

^d^Biological parent, federal state, current employment status, previous contact with social and family services, parental mental disorder, parental history of child abuse or neglect, children’s age, parental age, socioeconomic status, number of children in the household, and parental risk of alcohol abuse.

^e^Parental sex, biological parent, federal state, current employment status, previous contact with social and family services, parental mental disorder, parental history of child abuse or neglect, children’s age, parental age, socioeconomic status, and number of children in the household.

^f^Nationality: German, previous contact with social and family services, family or household member had a COVID-19 infection, family or household member was hospitalized due to COVID-19, parental chronic severe health condition, parental mental disorder, parent belongs to the risk group for severe COVID-19, parental history of child abuse or neglect, socioeconomic status, and parental risk of alcohol abuse.

^g^Parental sex, federal state, previous contact with social and family services, significant financial loss, parental chronic severe health condition, parental mental disorder, parental history of child abuse or neglect, children’s age, and parental risk of alcohol abuse.

^h^Parental sex, federal state, single parent, previous contact with social and family services, the availability of a garden, family or household member admitted to the hospital with COVID-19, significant financial loss, parental mental disorder, parental history of child abuse or neglect, parental experience of violence in adulthood, children’s age, parental age, number of children in the household, parental risk of alcohol abuse, and number of persons in the household.

^i^Parental sex, federal state, current employment status, previous contact with social and family services, significant financial loss, reduced working hours, job loss, parental mental disorder, parent belongs to the risk group for severe COVID-19, parental history of child abuse or neglect, parental experience of violence in adulthood, children’s age, socioeconomic status, child having another main place of residence, and parental risk of alcohol abuse.

^j^Parental sex, current employment status, previous contact with social and family services, significant financial loss, reduced working hours, job loss, parental mental disorder, parent belongs to the risk group for severe COVID-19, parental experience of violence in adulthood, children’s age, number of children in the household, and number of persons in the household.

**Table 6:** Comparison of the occurrence and frequency of adverse childhood experiences between the convenience sample and the specific representative sample.

		Occurrence										Increase during the COVID-19 pandemic						
		Values, n (%)		*χ*^*2*^ (*df*)		*P* value		φ		n (%)			*χ*^*2*^(*df*)		*P* value		φ	
**Severe stressful living conditions**					0.1 (1)		.75		0.004					0.4 (1)		.52		0.01
	Web-based sample	307 (6.2)								129 (2.6)								
	Representative sample	66 (6)								23 (2)								
**Physical abuse**					55.8 (1)		<.001		0.10					38.5 (1)		<.001		0.08
	Web-based sample	696 (14.1)								329 (6.6)								
	Representative sample	57 (5.6)								17 (2)								
**Nonverbal emotional abuse**					7.0 (1)		.03		0.03					10.5 (1)		.001		0.04
	Web-based sample	470 (10)								356 (7.1)								
	Representative sample	82 (8)								45 (4)								
**Supervisory neglect**					3.1 (1)		.08		–0.02					11.3 (1)		<.001		0.04
	Web-based sample		559 (11.3)							326 (6.6)								
	Representative sample		96 (9.4)							39 (4)								
**Emotional neglect**					24.7 (1)		<.001		0.06					0.8(1)		.37		–0.01
	Web-based sample		497 (10)							284 (5.7)								
	Representative sample		157 (15.3)							66 (6)								
**Verbal emotional abuse**					91.5 (1)		<.001		0.12					137.83		<.001		0.15
	Web-based sample		2423 (48.82)							1585 (31.91)								
	Representative sample		332 (32.4)							140 (13.7)								
**Witness of domestic violence**					107.9 (1)		<.001		0.13					116.91 (1)		<.001		0.14
	Web-based sample		2488 (50.22)							1236 (24.91)								
	Representative sample		332 (32.4)							97 (10)								
**Sexual abuse**					15.2 (1)		<.001		0.05					—^a^		—		—
	Web-based sample		19 (0.4)							2 (0.04)								
	Representative sample		14 (1.4)							3 (0.3)								
**Physical neglect**					8.4 (1)		.02		0.04					—		—		—
	Web-based sample		19 (0.4)							8 (0.2)								
	Representative sample		11 (1)							3 (0.3)								

^a^Inference statistics are only reported for cell counts ≥5 in all cells.

**Table 7:** Logistic regression analysis: investigating and explaining differences in the increase of child maltreatment outcomes between the convenience and the specific representative samples.

	Unadjusted			Adjusted		
	B (SE)	*P* value	OR^a^ (95% CI)	B (SE)	*P* value	OR (95% CI)
Verbal emotional abuse	1.08^b^ (0.10)	<.001	2.95 (2.44-3.56)	0.72^c^ (0.12)	<.001	2.05^d^ (1.63-2.57)
Witnessing domestic violence	1.16^e^ (0.11)	<.001	3.19 (2.56-3.97)	0.70^f^ (0.13)	<.001	2.02^g^ (1.55-2.63)
Nonverbal emotional abuse	0.50^h^ (0.16)	.002	1.65 (1.20-2.27)	0.53^i^ (0.20)	.01	1.70^j^ (1.15-2.52)
Emotional neglect	–0.14^k^ (0.14)	.33	0.87 (0.66-1.148)	–0.10^l^	0.19 (0.59)	0.90^m^ (0.62-1.30)

^a^OR: odds ratio.

^b^Model fit: *χ*^2^_1_=153.5; *P*<.001; Nagelkerke *R*^2^=0.04.

^c^Model fit: *χ*^2^_37_=589.6; *P*<.001; Nagelkerke *R*^2^=0.14; Hosmer and Lemeshow χ^2^_8_=10.3; *P*=.25.

^d^Covariates with a significant partial regression coefficient: Sample membership, parental sex, number of children in the household, parental age, children’s age, federal state, current employment status, previous contact with social and family services, the availability of a balcony, reduced working hours, job loss, parental mental disorder, parental history of child abuse or neglect, school and professional education, and child’s sex.

^e^Model fit: *χ*^2^_1_=137.1; *P*<.001; Nagelkerke *R*^2^=0.04.

^f^Model fit: *χ*^2^_37_=522.7; *P*<.001; Nagelkerke *R*^2^=0.13; Hosmer and Lemeshow *χ*^2^_8_=6.9; *P*=.54.

^g^Covariates with a significant partial regression coefficient: Sample membership, parental sex, number of children in the household, child’s age, single parent, material status, current employment status, previous contact with social and family services, significant financial loss, parental mental disorder, parental history of child abuse or neglect, and professional education: university.

^h^Model fit: *χ*^2^_1_=10.8; *P*=.001; Nagelkerke *R*^2^=0.01.

^i^Model fit: χ^2^_37_=248.6; *P*<.001; Nagelkerke *R*^2^=0.11; Hosmer and Lemeshow *χ*^2^_8_=11.1; *P*=.20.

^j^Covariates with a significant partial regression coefficient: Number of children in the household, children’s age, federal state, current employment status, previous contact with social and family services, the availability of a garden, significant financial loss, parental mental disorder, parental history of child abuse or neglect, parent belongs to the risk group for severe COVID-19, and professional education: apprenticeship.

^k^Model fit: *χ*^2^_1_=0.9; *P*=.33; Nagelkerke *R*^2^≤0.001.

^l^Model fit: *χ*^2^_37_=96.9; *P*<.001; Nagelkerke *R*^2^=0.05; Hosmer and Lemeshow *χ*^2^_8_=5.2; *P*=.74. Covariates with a significant partial regression coefficient.

^m^Covariates with a significant partial regression coefficient: Previous contact with social and family services, significant financial loss, and parental mental disorder.

#### Parental Stress

In January 2020, parents from the convenience sample reported significant but negligibly more parental stress (η^2^=0.003). However, at the time of the highest burden, parents from the convenience sample reported significantly higher parental stress than parents from the specific representative sample, with a small effect (η^2^=0.024).

#### Subjective Health

In January 2020, there was no significant difference (η^2^<0.001) between parents of the 2 samples regarding subjective health (η^2^<0.001). Again, at the time of the highest burden, parents from the convenience sample reported significantly poorer subjective health, with a small effect (η^2^=0.016).

#### Mental Health and General Stress

Parents in the convenience sample reported higher overall anxiety and depression symptoms (Patient Health Questionnaire-4) than parents in the specific representative sample at times of the highest burden, with a medium effect size (η^2^=0.055). The same pattern applied to general stress, with a medium effect (η^2^=0.044).

#### Pandemic-Related Stress

Samples did not differ in their overall pandemic-related stress (η^2^<0.001).

#### Child Maltreatment

In both samples, the lifetime occurrence of WDV (convenience sample: 2488/4966, 50.2% and specific representative sample: 332/1024, 32.42%) and VEA (convenience sample: 2423 4965, 48.82% and specific representative sample: 3321023, 32.4%) were the most frequent subtypes (Table 6). In the convenience sample, a higher share of parents reported the lifetime occurrence of VEA (φ=0.12), WDV (φ=0.13), NEA (φ=0.03), physical abuse (φ=0.10), and emotional neglect (φ=0.06). No differences were found for severe stressful living conditions, NEA, or supervisory neglect. The odds of a child experiencing an increase in one of the following subtypes of child maltreatment were higher in the convenience sample: for VEA (convenience sample: 1585/4966, 31.91% and specific representative sample: 140/1024, 13.7%), it was 2.95 times higher; for WDV (convenience sample: 1236/4961, 24.91% and specific representative sample: 97/1024, 9.47%), it was 3.19 times higher; and for NEA (convenience sample: 356/4966, 7.17% and specific representative sample: 45/1024, 4.39%), it was 1.65 times higher during the COVID-19 pandemic (Table 7). The odds (0.87) of an increase in the frequency of emotional neglect were not significantly different between the samples (convenience sample: 284/4967, 5.72% and specific representative sample: 66/1024, 6.45%).

### Explaining Differences in Parental Outcomes Between the 2 Samples

To explain differences in parental outcomes, we calculated ANCOVAs with sociodemographic data, parent-related risk factors, and COVID-19–related experiences as covariates. Only significant covariates are listed in Tables 5 and 7.

#### Parental Stress

At the time of the highest burden, the difference in parental stress between the samples remained significant but was negligible in size (η^2^=0.002) after controlling for sociodemographic data, parent-related risk factors, and COVID-19–related experiences. The covariates explained 92% of the difference in the outcome between the studies. The difference in parental stress between the samples in January was still not significant, and the effect size was negligible (η^2^<0.001). As there were no differences between samples, the covariates cannot explain any of them. It must be noted that the covariates also do not induce any relevant differences.

#### Subjective Health

The difference between the samples at the time of the highest burden was significant but negligible in size (η^2^=0.004) after controlling for sociodemographic data, parent-related risk factors, and COVID-19–related experiences. The covariates explained 75% (at the time of the highest burden) of the difference in outcome between the samples. The difference between the samples in January regarding subjective health was still not significant, and the effect size was negligible (η^2^<0.001). As there were no differences between samples, the covariates cannot explain any of them. It must be noted that the covariates also do not induce any relevant differences.

#### Mental Health and General Stress

The difference between the samples regarding mental health was small (η^2^=0.016) but significant after controlling for sociodemographic data, parent-related risk factors, and COVID-19–related experiences. The covariates explained 71% of the difference in the outcome between the samples. The same pattern applied to general stress (η^2^=0.014). In total, 68% of the difference in the outcome between the samples was explained by the covariates.

#### Pandemic-Related Stress

The difference between the samples regarding pandemic-related stress was significant, and the effect size slightly grew but was still negligible (η^2^=0.001) after controlling for sociodemographic data, parent-related risk factors, and COVID-19–related experiences. As there are no differences between studies, the covariates cannot explain any of them.

#### Child Maltreatment

There are large and significant differences in child maltreatment between the 2 samples in all subcategories except emotional neglect. The odds of experiencing VEA, WDV, and NEA were between 1.65 and 3.19 times higher in the convenience sample as compared to the specific representative sample. The difference in VEA and WDV between the samples could partly be explained by the covariates. After controlling for sociodemographic data, parent-related risk factors, and COVID-19–related experiences, the odds ratio of a child experiencing an increase in VEA and WDV in the convenience sample compared to the specific representative sample were 2.05 (as compared to 2.95 before adjustment) and 2.02 (as compared to 3.19 before adjustment), respectively. The differences in NEA between the samples could not be explained by the covariates. As there were no differences in emotional neglect between samples, there were no effect differences to be explained by the covariates. It must be noted that the covariates also do not induce any relevant differences.

## Discussion

### Principal Findings

So far, research has paid little attention to the representativeness of samples. However, this is crucial for drawing valid inferences on the population of interest. This is especially important in areas in which far-reaching decisions are made based on results of a study. This is specifically prevalent in studies on psychological consequences of the COVID-19 pandemic, which made immediate decisions necessary. In this paper, we showed that sampling strategy can have a great impact on the results of a study. We relied on data investigating parental outcomes in the COVID-19 pandemic of 2 web-based studies that differ in their sampling scheme, one being a typical convenience sample (N=4967) and the other a typical specific representative sample drawn by a survey institute (N=1024).

Our first aim was to investigate the impact of sampling strategy on the results. For this, we examined differences in results between the 2 samples. Our second aim was to investigate reasons for these differences. For this, we assessed (1) to what extent the studies differ in sample characteristics and to what extent the sample characteristics may explain group membership and (2) to what extent differences in parental outcomes between the 2 samples can be explained by sample characteristics (sociodemographic data, parent-related risk factors, and COVID-19–related experiences).

### Discussion of Results

#### Differences in Sample Characteristics

We found that the convenience sample differed from the specific representative sample in sociodemographic composition, parent-related risk factors, and COVID-19–related experiences. Sociodemographic data, parent-related risk factors, and COVID-19–related experiences predicted sample membership well. Thus, this study identified a set of variables that partly explain differences in study results and explain differences in the sample selection.

As expected, the sociodemographic composition of the 2 study samples (convenience and specific representative sample) differed in several ways (eg, overrepresentation of the female sex, younger parental and child age, a higher number of children, higher socioeconomic status, and higher education level in the convenience sample). The differences in the sociodemographic composition of the convenience sample were in line with previous findings [29-32,34-39]. Whitaker et al [33] also reported an overrepresentation of White, female individuals, and younger individuals with higher income and educational status compared to the general population in some studies in their literature review on Facebook recruitment. The overrepresentation of women in convenience samples is likely to be especially pronounced in studies related to mental and physical health [33,69] as well as parenting and family [35,38,80,92-94]. In various convenience samples evaluating parenthood during the COVID-19 pandemic, mothers constituted the majority [4,9,14-17,19,22,27,40-42,44,45]. However, the effects of this overrepresentation have not yet been empirically examined. Furthermore, in many of these convenience samples, most participants reported higher education [4,14,16,17,27,41,42,45-47]. One reason could be that women [95,96], younger people with younger children, and people with higher educational qualifications may more readily participate in web-based research and are thus more likely to be reached for study purposes [80,95,97]. Individuals with high educational attainment may be more familiar and more interested in research. In addition, the use of personal emails and invitations for recruitment may have resulted in a participant pool primarily from academic circles. The overrepresentation of women, along with younger parents and those with more children, could also be attributed to thematic self-selection. This group of parents, who were particularly stressed during the pandemic [4,14,59], may have been more motivated to share their experiences of hardship [30]. In summary, the sample composition of our convenience sample is comparable to many other samples recruited during the COVID-19 pandemic.

#### Differences in Parental Outcomes

Results on parental outcomes during the COVID-19 pandemic differed notably between the 2 samples. Overall, parents from the convenience sample were more burdened during the COVID-19 pandemic than parents from the specific representative sample. Compared with parents from the specific representative sample, parents from the convenience sample reported significantly more parental stress at the time of the highest burden, less subjective health, more anxiety and depression symptoms overall (Patient Health Questionnaire-4), more general stress, more often the occurrence of child maltreatment (VEA, WDV, physical abuse, NEA, and emotional neglect), and an increase of child maltreatment during the COVID-19 pandemic (VEA, WDV, and NEA). These results corroborate the claim that sampling has considerable impact on the results [31].

Our results are in line with findings from previous studies. Studies show that people with higher burdens are more likely to participate in web-based surveys than those with lower burdens [25,39,77,78]. The reasons may be a higher motivation to share one’s burden and a thematic awareness of the topic due to one’s own burden [30]. A higher amount of mental health burden in web-based studies with convenience sampling compared to representative sampling has also been observed in other studies with samples of parents [39,80] before the COVID-19 pandemic. Lawson et al [98] showed that the recruitment of web-based convenience samples influenced the prevalence of child abuse. Joyal-Desmarais et al [79] also found differences between samples in >70% of their variables when comparing their web-recruited convenience samples with representative surveys regarding preventive behaviors.

#### Explaining Differences in Parental Outcomes Between Studies

Differences in parental outcomes between the 2 samples could (partially) be explained by differences in sociodemographic data, parent-related risk factors, and COVID-19–related experiences between the samples. Consequently, at least for this study, we seem to have identified relevant confounders for the difference in effects between the 2 samples.

On the basis of our results, it is initially not possible to say which covariates are explicitly responsible for the differences. However, we can identify covariates that differed between the 2 samples: parental sex and age, children’s age, federal state (ie, city-state), school and professional education, number of children in the household, socioeconomic status, nationality, relationship status, biological parents, rooms and garden in the household, risk group of severe COVID-19, current or previous mental disorder, and history of own child abuse or neglect. Other studies identified sociodemographic variables as correlates for poorer mental health outcome (eg, female sex, younger parental and children age, parental psychological or physical health conditions before the COVID-19 pandemic, and lower financial background and income or financial stress) [8,11,16,40-42,48-60]. Furthermore, sociodemographic variables were also related to higher parental stress (eg, more children in the household, younger age of the children, and being a single parent) [4,14,17,20,41,46,59], and the occurrence of child maltreatment and parent-child conflicts (eg, younger parental and children age, job and financial loss, living in an urban area, and having low income or financial concerns) [1,2,43,46,47,59,98-100] for parents during the COVID-19 pandemic. Our convenience sample showed a higher presence of individuals with sociodemographic risks (such as more female individuals, younger age, and more and younger children), potentially contributing to greater disparities in parental outcomes due to an overrepresentation of these identified risk factors. Thus, these sociodemographic risk factors may explain differences in parental outcomes.

### Strengths

#### Investigation of the Impact of Sampling

So far, research has largely overlooked the impact of sampling on the informativeness of the results. With this paper, we present one of the few studies that highlight and evaluate the impact of sampling on the results of studies in clinical psychology.

#### Availability of 2 Studies With the Same Measurements

We were able to use 2 samples in which not only the same outcome measures but also the same explaining variables were measured in exactly the same way. This is a rather rare case, but it was essential for answering the research question. This also allowed us to identify variables explaining these differences. These factors could be assessed in future research on parental outcomes during pandemics to (1) understand which population groups a study provides information for and (2) for understanding differences in study results. Note that while we compared 2 typical sampling approaches—a convenience sample and a specific representative sample, as commonly used by survey institutes—this comparison provided insights into how these approaches lead to different results. However, this type of design and analysis is not limited to these specific sampling methods. The results of any sampling approaches, including 2 different convenience samples, can be compared in this way. The key is that the same variables are assessed in both studies.

#### Explaining Differences in Results

We not only investigated the differences in results between studies due to different sampling approaches but also aimed to understand why studies yield different results. The collection of a comprehensive set of explaining variables in both studies allowed us to explain most of the differences in study results. This is remarkable, as usually this is often not the case (eg, [31]). In many studies, only a few sociodemographic variables are available that are similarly assessed in the different studies. These usually cannot explain differences in results very well [101]. With sociodemographic data, parent-related risk factors, and COVID-19–related experiences, we had powerful explanatory variables for the differences in study results. If, as in many other studies, we had only limited sociodemographic data, we likely would have explained much less of the differences in the results.

#### Strategy for Investigating Differences in Results Across Studies

Our paper also presents a strategy for investigating reasons for differences in results across different studies. This strategy can also be applied to compare different sampling strategies in other fields. Thus, researchers may at the same time conduct studies with different types of sampling strategies. This may also be 2 different convenience samples. Relevant explaining variables need to be collected in the same way in both studies to analyze to what extent these can explain differences in outcomes between the 2 studies.

### Limitations and Outlook

#### Limitations of the Comparison of the Studies

Our aim was to investigate the impact of the sampling strategy on the study results on parental outcomes. While we could make use of the rare case of having 2 studies with different sampling strategy that were almost identical in their design and setup, not all design aspects were identical. First, while participants in the convenience sample were interviewed via web surveys (CAWI), parents in the specific representative sample were interviewed either via CAWI or CATIs. This could have led to differences in response behavior, especially with regard to sensitive topics (eg, adverse childhood experiences and parental mental disorder) due to social desirability. Second, the 2 studies partly differed with respect to the incentives they received. Parents in the convenience sample and parents in the specific representative study that were interviewed via CAWI may have been particularly motivated by the prospect of incentives (money, vouchers, and Ipad). In contrast, parents in the specific representative study that were interviewed via telephone did not receive any incentives. This could have also influenced response behavior, leading to differences in study results. In summary, in addition to differences in the sample composition, there are further explanations for differences in the results between the samples, such as different modes of data collection in the representative sample (CATI and CAWI) versus the convenience sample (only CAWI) and different motivations of participants (no incentives for participants in CATI in the quota sample vs incentives for participants in CAWI and the convenience sample). These were not controlled for and must be considered in the final evaluation of the study results. While we could explain a large part of the differences in results between the 2 studies, some unexplained differences remained. These may be due to unobserved explanatory variables, other factors that could account for both sample differences and variations in parental outcomes, or differences in the study designs.

From our study, we cannot necessarily conclude that the specific representative sample results in more valid conclusions on the underlying population than the convenience sample. If selection bias is stronger on relevant variables not controlled for in the specific representative sample as compared to the convenience sample, bias could even be larger in the specific representative study. What we surely can conclude is that the sampling strategy does have a large impact on the study results. For the specific representative study, we can rule out that sample selection due to strata variables biases the results in this study.

#### Limitations of Single Studies

While both studies were conducted rigorously with the aim of accurately inferring parental outcomes in the population, each study has its own limitations. First, while the 2 studies aimed at drawing inferences on parents with underaged children in Germany, due to practical reasons, they had to restrict the sample to German-speaking parents, which excludes a part of the population [102]. Second, although anonymity was assured by different measures, we cannot rule out that participants answered in a socially desirable way, refraining from reporting, for example, child maltreatment. Though we tried to reduce this, it needs to be kept in mind when interpreting the results. All data were collected and evaluated anonymously. The researchers did not have the contact details of the participants available at any time. This was also explained to the participants in the informed consent form. Overall, our lifetime prevalence rates of child maltreatment are in line with the prevalence rates found in the literature [103]. Third, neither of the 2 samples is globally representative of the general population and is, thus, limited in generalizing to the overall population. While specific representativeness for some variables can be assured for the specific representative study, selection bias may still exist for all other variables. Note that in this specific setting, it would have been possible to weight the convenience sample using census data, thereby achieving representativeness for this sample as well. As compared to the quota sample, specific representativity in the convenience sample would be solely achieved by poststratification. As our aim was to investigate the impact of sampling on study results, we did not consider this here.

Even if their results cannot necessarily be generalized to the underlying population, both the specific representative as well as the convenience samples may provide valuable insights if the sample is well described with respect to variables relevant to the construct under investigation. Adding up information from many studies (that may all be convenience samples) does help to build theory and to describe for which type of persons the theory has been tested for. For this, it is important that relevant person characteristics of the sample are comprehensively described. These should go beyond just some demographic variables and should consider the variables that are relevant to the phenomena of interest. Further, if we know the distribution of these variables in the population, we can infer population-level results from a convenience sample (eg, by weighting the observations in the convenience sample). This approach is similar to how election results are predicted [104].

#### Generalizability of Our Results

As with any research study, it is not possible to generalize findings from a single investigation to all other studies. Therefore, the results presented in this paper may not necessarily apply to other studies in clinical psychology or those focused on parental outcomes. As in other research, our study provides another piece on the topic that together with other studies adds up to a broader picture. We presume that the results of our study also hold for studies that use similar outcomes to those of our study and recruitment or sampling strategies that do not differ massively from ours. The results of this study may not necessarily be the same for other research topics (specifically other outcomes) and other sample selection mechanisms. In a different study, the selection mechanism of participants in the sample may be different, which leads to different results and possibly also different explaining variables that explain the differences. Different research questions within a study lead to different outcome measures. These are most likely impacted by different explaining variables (covariates). While it is not possible to generalize the findings of this study to other studies, this study shows (1) that sampling strategy can have a large impact on the results and (2) how one can investigate reasons for differences in the results. Relevant covariates that explain differences in study results will most probably be those that are strongly related to the outcomes (researchers usually have a good theory about that from the literature in their field) as well as are relevant for participating in a study (research on this is usually a bit scarce, but pilot studies may help). Assessing these variables as covariates is a promising strategy as it leaves less room for unexplained variation in the outcome [101]. Note that this is not tied to having a quota (ie, a specific representative) sample; it can be done with any 2 studies. The requirement is that the same covariates are assessed in both studies. Future studies may want to incorporate this into their study planning.

### Conclusions

To allow readers to judge the representativity of a study, detailed information on sampling and recruitment as well as on generalizability should be provided (eg, constraints on generality [105]).

Our results show that the results of studies differ depending on the sampling strategy. We were able to investigate this. Scientists need to be aware of the fact that from a single study, one cannot directly generalize to the population of interest. Full (global) representative studies are the gold standard in research. However, these are often not feasible for various reasons. Therefore, scientists should collect as many relevant explaining variables (covariates) as possible to describe the sample selection. Such variables are usually those that may have an impact on the results. Collecting these variables makes it possible to understand for which subgroups in the population conclusions can be drawn. As our study shows, if the same explaining variables are assessed in different studies, it is also possible to explain differences in the study results by differences in the group characteristics.

Even if researchers just perform a single study with a specific sampling strategy, we recommend collecting data on potentially explaining variables. This (1) allows to judge for which part of the population the results hold and (2) may allow future studies that assess the same explaining variables to explain differences in their results. If researchers even have information on the distribution of these variables in the general population, they may adjust for sampling bias using sampling weights [31] (eg, [12,13,106]). The choice of explanatory variables is crucial for this.

If possible, scientists may want to consider assessing explaining variables and covariates that are (1) relevant to the outcome under consideration and (2) possibly related to the selection into the study. These will help to identify which piece of the puzzle is actually added to the overall theory by this study. It also helps to integrate results over multiple studies in the future. If they have information on the distribution of these variables in the target population, they may even draw a quota sample or weight their convenience sample to assure specific representativeness. In most cases, it will not be possible to assess all relevant explaining variables. However, considering some relevant explanatory variables can already enhance the robustness of the results.

## Appendix

Multimedia Appendix 1 The flyer on the recruitment of the convenience sample, a table about the sample composition regarding the Federal State of Germany of the web-based convenience sample and the representative sample, and a detailed table about the logistic regression analysis to predict sample membership through our sociodemographic data, parent-related risk factors, and COVID-19–related experiences.

## Abbreviations

ANCOVA: analyses of covariance
CATI: computer-assisted telephone interview
CAWI: computer-assisted web interview
NEA: nonverbal emotional abuse
VEA: verbal emotional abuse
WDV: witnessing domestic violence

## Data Availability

The datasets generated and analyzed during this study are available from the corresponding author upon reasonable request.
