# Supplementary material for: Parental Mental Health and Child Maltreatment in the COVID-19 Pandemic: Importance of Sampling in a Quantitative Statistical Study
Source: J Med Internet Res. 2025 Jan 24;27:e52043. doi: 10.2196/52043 (PMC11806267; doi:10.2196/52043)
Supplement: Multimedia Appendix 1 [file jmir_v27i1e52043_app1.docx]

**Multimedia Appendix**


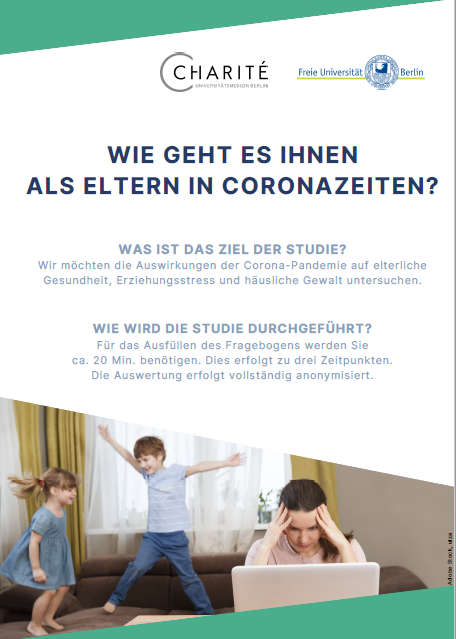


English translation:

HOW ARE YOU DOING AS PARENTS IN COVID TIMES? WHAT IS THE AIM OF THE STUDY? We want to investigate the effects of the COVID pandemic on parental health, parenting stress and domestic violence. HOW IS THE STUDY CONDUCTED? To complete the questionnaire you will need approx. 20 minutes. This will be done at three points in time. The evaluation will be completely anonymized.


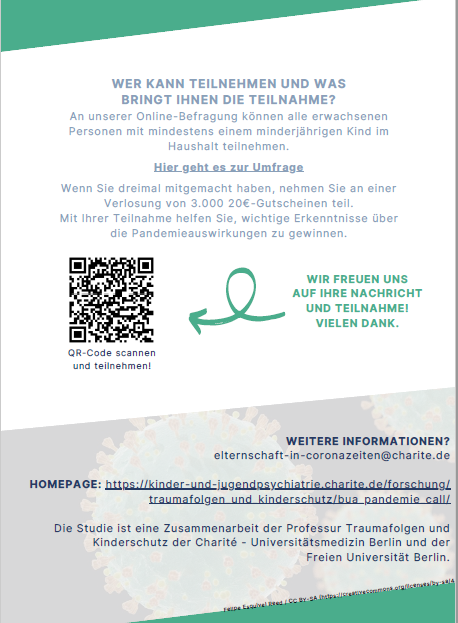


Picture 1. Flyer on the recruitment of the convenience sample

English translation:

WHO CAN PARTICIPATE AND WHAT DO THEY GAIN FROM TAKING PART? Our online survey is open to all adults with at least one underage child living in the household can take part. Click here for the survey. If you have taken part three times, you will be entered into a prize draw for 3,000 €20 vouchers. By taking part, you will help to gain important insights into the effects of the pandemic. Scan the QR code and take part! WE LOOK FORWARD TO YOUR MESSAGE AND PARTICIPATION! THANKS A LOT. MORE INFORMATION? elternschaft-in-coronazeiten@charite.de . HOMEPAGE: https: https://kinder-und-jugendpsychiatrie .charite.de/forschung/traumafolgen_und_kinderschutz/bua_pandemie_call/ The study is a collaboration between the Chair of Trauma Consequences and Child Protection at Charité -Universitätsmedizin Berlin and the Freie Universität Berlin. WE LOOK FORWARD TO YOUR MESSAGE AND PARTICIPATION! THANK YOU.

Table S1

Sample composition regarding the Federal State of Germany of the online convenience sample and the representative sample

|  | **online sample 2020**  **N=4967**  **n (%)** | **representative sample 2020^a^**  **N=1024**  **n (%)** |
| --- | --- | --- |
| Baden-Württemberg | 358 (7.2%) | 134 (13.1%) |
| Bayern | 468 (9.4%) | 160 (15.6%) |
| Berlin | 2111 (42.5) | 44 (4.3%) |
| Brandenburg | 186 (3.7%) | 32 (3.1%) |
| Bremen | 28 (0.6%) | 8 (0.8%) |
| Hamburg | 124 (2.5%) | 22 (2.2%) |
| Hessen | 270 (5.4%) | 77 (7.5%) |
| Mecklenburg-Vorpommern | 46 (0.9%) | 21 (2.0%) |
| Niedersachsen | 305 (6.1%) | 98 (9.6%) |
| Nordrhein-Westfalen | 617 (12.4%) | 222 (21.6%) |
| Rheinland-Pfalz | 116 (2.3%) | 50 (4.9%) |
| Saarland | 23 (0.5%) | 13 (1.2%) |
| Sachsen | 121 (2.4%) | 52 (5.1%) |
| Sachsen-Anhalt | 40 (0.8%) | 29 (2.8%) |
| Schleswig-Holstein | 96 (1.9%) | 36 (3.5%) |
| Thüringen | 58 (1.2%) | 28 (2.7%) |

Table S2

Logistic regression analysis to predict sample membership through our sociodemographic data,

parent-related risk factors and COVID-19 related experiences

|  | **B** | **SE** | ***P*** | **OR** | **95% CI OR** | |
| --- | --- | --- | --- | --- | --- | --- |
|  |  |  |  |  | **Lower** | **Upper** |
|  |  |  |  |  |  |  |
| **Parental gender female** | **2.410** | **0.115** | **p < .001** | **11.133** | **8.892** | **13.940** |
| Number of Persons in household | 0.026 | 0.100 | 0.797 | 1.026 | 0.843 | 1.249 |
| **Number of children in houshold** | **0.266** | **0.115** | **0.021** | **1.305** | **1.042** | **1.634** |
| **parental age** | **0.045** | **0.009** | ***P* < .001** | **1.046** | **1.027** | **1.065** |
| rooms | -0.016 | 0.011 | 0.160 | 0.984 | 0.963 | 1.006 |
| Socioeconomic status | -0.001 | 0.004 | 0.868 | 0.999 | 0.992 | 1.007 |
| **children age** | **-0.201** | **0.015** | ***P* < .001** | **0.818** | **0.794** | **0.842** |
| nationality: german | -0.175 | 0.336 | 0.602 | 0.839 | 0.435 | 1.621 |
| **Biological parent** | **0.718** | **0.314** | **0.022** | **2.051** | **1.109** | **3.793** |
| **Federal State of Germany: city state** | **2.691** | **0.153** | ***P* < .001** | **14.750** | **10.929** | **19.906** |
| Single parent | -0.176 | 0.277 | 0.526 | 0.839 | 0.487 | 1.445 |
| **Marital status: in a relationship** | **0.622** | **0.271** | **0.022** | **1.863** | **1.094** | **3.170** |
| Current employment status: working | 0.146 | 0.132 | 0.270 | 1.157 | 0.893 | 1.498 |
| **Others in household** | **-0.522** | **0.220** | **0.017** | **0.593** | **0.386** | **0.912** |
| Previous contact to social and family services | 0.006 | 0.118 | 0.960 | 1.006 | 0.798 | 1.268 |
| **Balcony** | **-0.253** | **0.100** | **0.011** | **0.776** | **0.638** | **0.945** |
| Garden | -0.143 | 0.120 | 0.235 | 0.867 | 0.685 | 1.097 |
| Family/household member infected with COVID-19 | -0.508 | 0.366 | 0.165 | 0.602 | 0.294 | 1.232 |
| Family/household member admitted to hospital with COVID-19 | 0.412 | 0.873 | 0.637 | 1.509 | 0.273 | 8.350 |
| Family/household member died with COVID-19 | -0.026 | 0.908 | 0.977 | 0.974 | 0.164 | 5.768 |
| Reduced working hours | 0.126 | 0.116 | 0.274 | 1.135 | 0.905 | 1.423 |
| Job loss | -0.303 | 0.236 | 0.198 | 0.738 | 0.465 | 1.172 |
| **Significant financial loss** | **0.277** | **0.132** | **0.036** | **1.320** | **1.018** | **1.710** |
| Parental chronic severe health condition | 0.073 | 0.227 | 0.748 | 1.076 | 0.689 | 1.678 |
| **Parental mental disorder** | **0.780** | **0.149** | ***P* < .001** | **2.182** | **1.628** | **2.923** |
| Parental experience of violence in adulthood | -0.136 | 0.153 | 0.375 | 0.873 | 0.647 | 1.178 |
| **Parental history of child abuse or neglect** | **0.536** | **0.114** | ***P* < .001** | **1.709** | **1.366** | **2.138** |
| Parent belongs to risk group for severe COVID-19 | -0.030 | 0.270 | 0.913 | 0.971 | 0.572 | 1.648 |
| **another center of life** | **-0.876** | **0.301** | **0.004** | **0.416** | **0.231** | **0.751** |
| **School eduacation: Low (up to 9 years of schooling)** | **-1.457** | **0.522** | **0.005** | **0.233** | **0.084** | **0.648** |
| School eduacation: Middle (10 years of schooling) | -0.828 | 0.490 | 0.091 | 0.437 | 0.167 | 1.143 |
| **School eduacation: High (up to 13 years of schooling)** | **0.696** | **0.489** | **0.155** | **2.005** | **0.769** | **5.226** |
| Professional education: apprenticeship | -0.186 | 0.202 | 0.357 | 0.830 | 0.558 | 1.234 |
| Professional education: technical school | 0.102 | 0.239 | 0.669 | 1.108 | 0.693 | 1.771 |
| **Professional education: university (of applied science)** | **0.704** | **0.215** | **0.001** | **2.022** | **1.326** | **3.082** |
| gender first child | -0.050 | 0.094 | 0.596 | 0.951 | 0.791 | 1.144 |
| constant | -1.874 | 0.799 | 0.019 | 0.153 |  |  |

Notes. df = 1, significant predictors are bold. Model fit: χ²(36) = 2411.38, *P* < .001, Nagelkerke’s R² = 0.56,

Hosmer and Lemeshow χ²(8) = 8.0, *P* = .44.

This is a Multimedia Appendix to a full manuscript published in the J Med Internet Res. For full copyright and citation information see http://dx.doi.org/10.2196/jmir.52043
